# DC-SIGN Mediated Sphingomyelinase-Activation and Ceramide Generation Is Essential for Enhancement of Viral Uptake in Dendritic Cells

**DOI:** 10.1371/journal.ppat.1001290

**Published:** 2011-02-17

**Authors:** Elita Avota, Erich Gulbins, Sibylle Schneider-Schaulies

**Affiliations:** 1 Institute for Virology and Immunobiology, University of Würzburg, Wuerzburg, Germany; 2 Department of Molecular Medicine, University of Essen, Essen, Germany; The Salk Institute for Biological Studies, United States of America

## Abstract

As pattern recognition receptor on dendritic cells (DCs), DC-SIGN binds carbohydrate structures on its pathogen ligands and essentially determines host pathogen interactions because it both skews T cell responses and enhances pathogen uptake for *cis* infection and/or T cell *trans*-infection. How these processes are initiated at the plasma membrane level is poorly understood. We now show that DC-SIGN ligation on DCs by antibodies, mannan or measles virus (MV) causes rapid activation of neutral and acid sphingomyelinases followed by accumulation of ceramides in the outer membrane leaflet. SMase activation is important in promoting DC-SIGN signaling, but also for enhancement of MV uptake into DCs. DC-SIGN-dependent SMase activation induces efficient, transient recruitment of CD150, which functions both as MV uptake receptor and microbial sensor, from an intracellular Lamp-1+ storage compartment shared with acid sphingomyelinase (ASM) within a few minutes. Subsequently, CD150 is displayed at the cell surface and co-clusters with DC-SIGN. Thus, DC-SIGN ligation initiates SMase-dependent formation of ceramide-enriched membrane microdomains which promote vertical segregation of CD150 from intracellular storage compartments along with ASM. Given the ability to promote receptor and signalosome co-segration into (or exclusion from) ceramide enriched microdomains which provide a favorable environment for membrane fusion, DC-SIGN-dependent SMase activation may be of general importance for modes and efficiency of pathogen uptake into DCs, and their routing to specific compartments, but also for modulating T cell responses.

## Author Summary

Dendritic cells (DCs) bear receptors specialized on recognition of patterns specific to pathogens (such as carbohydrates), which can either promote functional activation of these cells (such as TLRs), which renders them capable of efficiently presenting antigens to T cells, or, as DC-SIGN, endocytic uptake as essential for loading MHC molecules. Viruses such as HIV and measles virus (MV) exploit DC-SIGN for both their uptake into DCs and modulation of TLR signaling, yet how this is mechanistically exerted is poorly understood. We now show that DC-SIGN activates sphingomyelinases (SMases) which convert their sphingomyelin substrate into ceramides, thereby catalysing the formation of membrane platforms able to recruit and concentrate receptors and associated signaling components. We found DC-SIGN-dependent SMase activation as essential for DC-SIGN and thereby modulation of TLR signaling, but also for enhancement of MV uptake. This is mediated by a fast, transient recruitment of its entry receptor, CD150, from an intracellular storage compartment to the cell surface where it co-clusters in ceramide enriched platforms with DC-SIGN. The ability to segregate viral receptors into (or exclude them from) membrane microdomains, which, based on their biophysical properties, facilitate membrane fusion, proposes DC-SIGN-mediated SMAse activation as a central regulator of pathogen uptake into DCs.

## Introduction

Their interaction with myeloid dendritic cells (DCs) is believed to be central to the understanding of immunomodulation by viruses also including measles virus [1], [2], [3], [4]. In the hematopoetic system, MV replication segregates with expression of CD150, an Ig-like domain containing molecule, expression of which is usually low on lymphocytes and immature DCs, where it is upregulated on activation by TLR ligation or inflammatory stimuli [5], [6], [7]. CD150 is sufficient to support MV binding, fusion and cell entry *in vitro* and *in vivo* [8]. In DCs, in common with other viruses, DC-SIGN enhances entry, and this is important in viral spread to secondary lymphatics and transmission to T cells, but also for modulation of DC viability and function and thereby determine T cell activation in quantitative and qualitative terms [9], [10], [11], [12], [13].

DC-SIGN is a C-type lectin receptor which functions to regulate adhesion by interaction with integrins, but also, as a pattern recognition receptor (PRR), to recognize carbohydrate structures on pathogens, thereby targeting them for endocytic uptake, processing and subsequent presentation [13], [14], [15]. It is enriched in nanoclusters at the leading edge on the DC plasma membrane, where ligands are acquired and then transported rearward to mid-lamellar sites for subsequent endocytosis [16], [17], [18], [19]. On differential recognition of carbohydrates, DC-SIGN signals and its signalosome involves a scaffolding complex containing lymphocyte specific protein 1 (LSP1), kinase suppressor of Ras1 (KSR1) and connector enhancer of ksr (CNK) as required for Raf-1 recruitment [20]. DC-SIGN-induced Raf-1 kinase activation was linked to modulation of TLR signaling at the level of NF-κB activation by promoting activation of its p65 subunit and thereby increasing initiation and duration of cytokine gene transcription [11], [21], [22].

By unknown mechanisms, viruses can escape lysosomal degradation thereby avoiding immune surveillance, and rather exploit DC-SIGN to gain entry to DCs [12], [13], [23], [24]. Similarly, how DC-SIGN enhances viral uptake for infection (referred to as ‚cis-infection') or internalization into and storage in non-lysosomal compartments for subsequent transfer to conjugating T cells (referred to as ’trans-infection') is mechanistically not well understood, however, co-segregation or concentration of virions or their respective low level expressed uptake receptors has been proposed to contribute [1], [25].

Local enrichment of ceramides is known to promote biophysical alterations of the membrane which can support fusion and negative curvature, but also segregation of membrane receptors and signalosome components thereby regulating a large variety of cellular processes [26], [27], [28], [29]. In response to a variety of stimuli also including ligation of TNF-R family members and Fcγ receptors, neutral and acid sphingomyelinases (SMases: NSM or ASM) are activated to generate membrane ceramides, which, on ASM activation, cause formation of outer membrane ceramide–enriched platforms [30], [31], [32]. In contrast to NSM, ASM is compartimentalized in non-lysosomal vesicles from where, on activation, it is recruited to the cell surface to catalyze breakdown of sphingomyelin (SM) into phospho-choline and ceramide. Ceramides act to convey and modulate receptor signaling by segregating or concentrating signaling components and this also includes KSR1, which catalyzes c-Raf-1 activation thereby enhancing its activity towards ERK1/2 [33], [34], [35], [36], [37]. As they promote receptor clustering and formation of membrane invaginations, ceramides can enhance endocytic uptake of viruses entering their target cells by this route [38], [39]. Ceramides can, however, also enhance intracellular vesicle fusion [40]. Thus, regulation of lateral segregation and concentration of receptors by ceramide-enriched platforms (or interference with that, as evidence for HIV [40] and of membrane fusion may be key to understanding the role of ceramides in viral uptake.

We now show that DC-SIGN ligation causes transient activation of both NSM and ASM within 3 to 15 mins. and this is accompanied by membrane ceramide accumulation. DC-SIGN signaling accounting for c-Raf-1 and ERK activation is abrogated on pharmacological interference with ASM activation indicating that activation of this enzyme is essential in this process. SMase activation also accounted for enhancement of MV uptake into DCs and this was promoted by DC-SIGN dependent surface recruitment of the MV binding and uptake receptor CD150, which was surface recruited from an intracellular storage compartment containing ASM. These data, for the first time, describe and mechanistically link regulated membrane lipid dynamics to modulation of PRR-dependent uptake into DCs, which may be relevant for viral and general entry processes into these cells.

## Results

### DC-SIGN ligation promotes ceramide accumulation on DCs in a SMase-dependent manner

**Figure 1:**
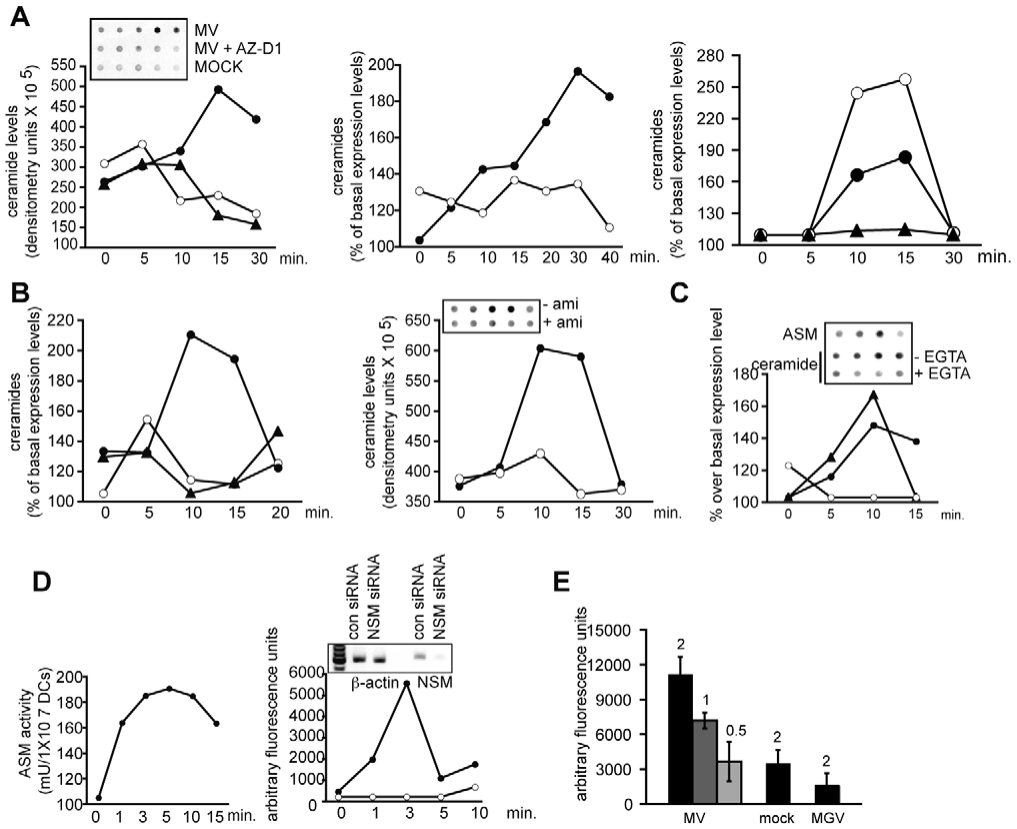
DC-SIGN ligation activates SMases and causes membrane ceramide accumulation on DCs. A. to C. Surface ceramide levels on DCs were determined by spot assays (shown in the insets) or flow cytometry (A, B). A. DCs were exposed to a MOCK preparation (open circles left panel), MV alone (black circles, left and middle panels) or after pretreatment with a blocking DC-SIGN antibody (AZ-D1) (black triangles, left panel) or in the presence of EGTA (open circles, middle panel). Right panel: DCs were left untreated (black circles) or exposed to an αCD150-antibody (5C6) prior to MV or MOCK treatment (white circles and black triangles, respectively). B. DCs were exposed to AZ-D1 alone (open circles, left panel) or after addition of a crosslinking antibody (filled circles, both panels). DCs were stimulated with isotype antibody as a negative control (black triangles, left panel) or DC-SIGN was ligated after 2 hrs pretreatment with amitriptyline (open circles, right panel). C. DCs were exposed to mannan for the time intervals indicated and surface levels of ASM (black triangles) and ceramides in the absence (black circles) or presence of EGTA (open circles) were analysed by spot assays (left panel). D. Whole cell lysates (for ASM, left panel) or membrane fractions (for NSM, right panel) of DCs exposed to mannan for the time intervals indicated were used to determine enzyme activities using a commercial kit. DCs were transfected with NSM siRNA (open circles) or not (black circles) (right panel, with effciency of NSM ablation shown by RT-PCR in the inset). E. ASM activity was determined 10 mins following exposure of DCs to MV or a recombinant MV expressing VSV G protein instead of the MV glycoproteins (MGV) at the multiplicities of infection indicated or a mock preparation applied at a concentration corresponding to m.o.i 2) as in D. Experiments shown are representatives of each three independent experiments involving different donors.

Membrane ceramide platforms segregate receptors and signalosomes both of which can affect viral entry. DC-SIGN may act to trap or concentrate virions (also including MV) for receptor interaction, and we thus analysed whether MV interaction with this molecule promoted membrane ceramide accumulation on DCs by employing an assay based on immunodetection of an a-ceramide antibody bound to intact cells (spot assay). On MV exposure, DCs responded by an about twofold increase in extrafacial ceramides which peaked at 15 mins and subsequently returned to baseline levels (Fig. 1A, left panel). Ceramide accumulation occurred DC-SIGN dependently, since it was efficiently abrogated upon pre-exposure of DCs with a DC-SIGN-specific antibody (AZ-D1) or EGTA, which prevents Ca2+-dependent DC-SIGN ligand binding (Fig. 1A, left panel and middle panel). In contrast, antibodies blocking MV interaction with its entry receptor, CD150, did not prevent, yet even slightly enhanced MV ceramide induction (Fig. 1A, right panel). Similar to MV, the DC-SIGN binding antibody AZ-D1 increased membrane ceramide display when crosslinked, while this was not observed with the antibody alone nor an isotype control antibody alone (Fig. 1B, left panel; for further experiments, DC-SIGN-specific antibodies were thus used crosslinked). Ceramide production in response to DC-SIGN ligation was sensitive to the ASM inhibitor amitriptyline indicating that activation of this enzyme was involved (Fig. 1B, right panel). To assess activation of SMases directly, we determined their activity after exposure of DCs to mannan, a well-defined DC-SIGN agonist. In line with amitriptyline sensitivity of ceramide generation, ASM surface display raised about 1.8 fold almost immediately following mannan addition, and this was paralleled by a rise in extrafacial ceramides both of which were EGTA sensitive (Fig. 1C, and not shown). Mannan-dependent activation of ASM was further confirmed using a commercial detection assay (Fig. 1D, left panel) which essentially mirrored kinetics and magnitude of the response determined by spot assays. Using the same experimental approach, a rapid, very efficient activation of NSM was also measured (about 5-fold) which peaked after 3 mins and then vanished, and this was entirely prevented upon RNAi mediated silencing of NSM expression (Fig. 1D, right panel). Importantly, ASM activation also occurred on MV exposure of DCs in a dose dependent manner, and this relied on the presence of the MV glycoproteins because it was not observed when a recombinant MV expressing the VSV G protein instead was used (Fig. 1E). Altogether these findings indicate that ligation of DC-SIGN by antibodies, mannan or MV promotes rapid activation of SMases, and ASM-dependent ceramide accumulation in the outer membrane leaflet.

### ASM activation is essential for DC-SIGN signaling

**Figure 2:**
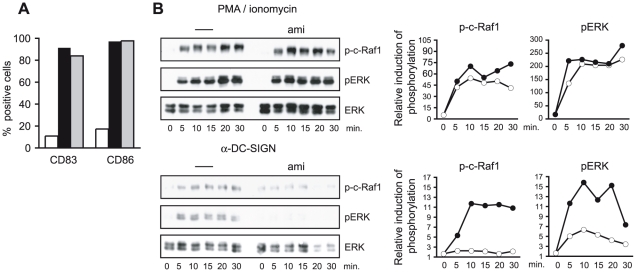
ASM activity is essential for DC-SIGN signaling. A. Surface expression levels of CD83 and CD86 were determined on DCs left untreated (iDCs, white bars) or pre-exposed to amitriptyline (black bars) or not (grey bars) prior to LPS stimulation (mDCs) after 24 hrs by flow cytometry. B. Levels of p-c-Raf1 and p-ERK were determined in DCs left untreated (right graphs: each black circles) or exposed to amitriptyline for 2 hrs (right graphs: each open circles) and subsequently activated by PMA/ionomycin (upper panels and right graphs, obtained after densitometric quantification) or AZ-D1 (followed by crosslinking) for the time intervals indicated (bottom panels and right graphs). ERK detection served as loading control.

**Figure 3:**
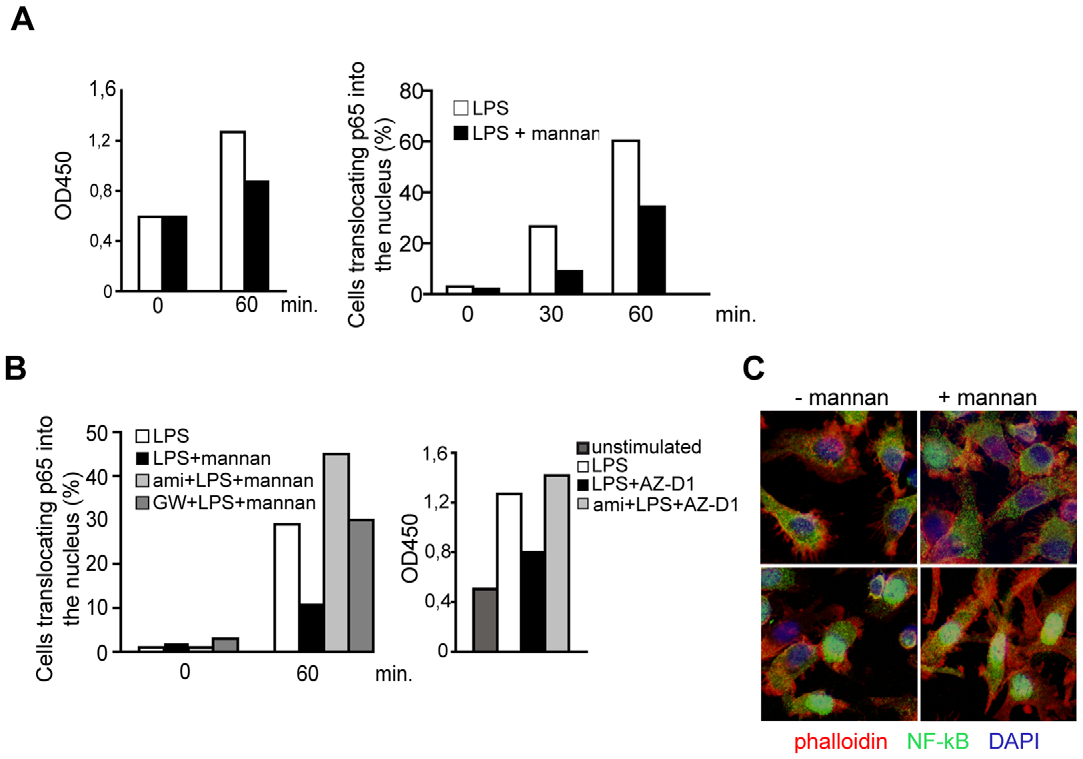
SMase dependent DC-SIGN signaling dampens rather than enhances TLR-stimulated NF-κB activation. A. N
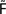
κB activation in DCs activated by LPS alone (white bars) or in the presence of mannan (black bars) was determined by measuring DNA binding using an ELISA based kit (left panel) or by determining the percentage of cells translocating p65 to the nucleus (right panel; 100 cells per treatment were recruited into the analysis, for an example see 3C) after the time intervals indicated. B. N
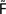
κB activation in DCs exposed LPS alone (white bars) or together with mannan only (left panels, black bars) or after a 2 hrs pretreatment with amitriptyline (left panel, light grey bars) or to GW4869 (left panel, dark grey bars) was analysed by determining the percentage of cells translocating p65 to the nucleus (each 150 cells per treatment were recruited into the analysis), or in DCs left untreated (right panel dark grey bar) or activated for one hour by LPS alone (right panel, white bar) or together with AZ-D1 (followed by crosslinking) only (right panel, black bar) or after a 2 hrs pre-treatment with amitriptyline (right panel, light grey bar) by measuring p65 DNA binding by ELISA. C. Representative images showing examples for scoring into nuclear and cytoplasmic localization as evaluated in A and B. Upper left panel: −LPS, upper right and bottom left panels: +LPS, lower right panel: ami +LPS. Cells were counterstaining by phalloidin (mainly detecting cortical f-actin lining the plasma membrane) and DAPI. Data shown in A–C represent each one representative out of three independent experiments.

DC-SIGN signaling includes activation of c-Raf-1 and ERK [11], [22], [41]. To asses if DC-SIGN signaling involves SMase activation, cells were pre-exposed to amitriptyline which per se did not affect DC viability (not shown) or LPS-induced upregulation of CD86 or CD83 after 24 hrs (Fig. 2A). PMA/ionomycin-dependent activation of c-Raf-1 or ERK as determined by detection of p-c-Raf-1 or pERK within 30 mins was unaffected on pre-exposure of DCs to amitriptyline (Fig. 2B, upper panels). In line with earlier findings obtained upon ManLam-, antibody or HIV exposure [11], [22], [41], DC-SIGN ligation by crosslinked AZ-D1 caused c-Raf-1 and ERK activation (Fig. 2B, bottom panels). In contrast to that induced by PMA/ionomycin, however, α-DC-SIGN induced c-Raf-1 and ERK phosphorylation was sensitive to amitriptyline indicating that DC-SIGN signaling involves ASM activation (Fig. 2B, bottom panels). DC-SIGN signaling does not confer NF-κB activation, yet apparently modulates that induced upon TLR ligation [11]. As revealed both by an ELISA kit based detection system or nuclear translocation of p65, TLR4 ligation by LPS indeed promoted NF-κB activation after 60 mins. (Fig. 3A). Mannan exposure did, however, reduce magnitude of NF-κB activation measured by either method indicating that DC-SIGN ligation interferes with TLR4 signaling (Fig. 3A and B). Interestingly, however, ablation of DC-SIGN signaling by amitriptyline or an NSM inhibitor, GW4896, apparently enhanced LPS-induced NF-κB activation as reflected by efficient nuclear accumulation of p65, and enhanced levels of activation as determined by ELISA (Fig. 3B and C). Overall, these findings support the interpretation that DC-SIGN membrane signaling essentially involves ceramide generation, and may act to dampen rather than to enhance TLR-dependent NF-κB activation and thereby production of pro-inflammatory cytokines.

### SMase activation enhances MV infection of DCs

**Figure 4:**
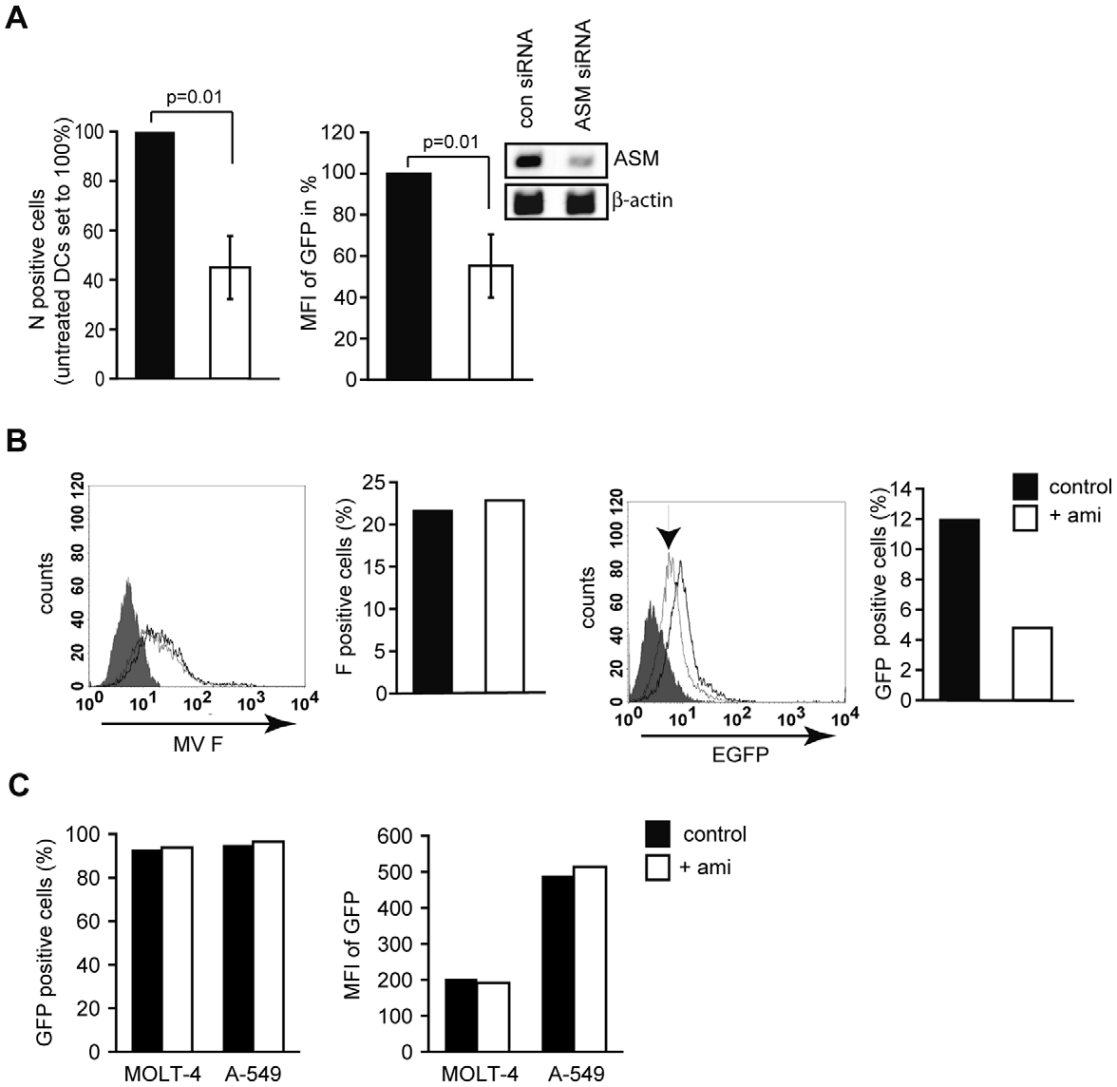
SMase activation enhances MV infection of DCs. A. Left panel: DCs were left untreated (black bar) or exposed to amitriptyline (white bar) prior to MV infection. Right panel: An GFP-tagged MV was used for infection of DC cultures where expression of ASM had been siRNA silenced (white bar) or not (black bar) (inset shows efficiency of silencing by RT-PCR). The frequency of cells expressing the MV N protein (left panel) or GFP (right panel) was determined after 12 hrs by flow cytometry. Values shown were generated in each three independent experiments involving different donors, p-values are indicated. B. DCs left untreated (each black bars and black lines) or pre-exposed to amitriptyline (each white bars and grey lines) were exposed to MV and virus binding (after 2 hrs at 4°C, left panel and graph) or uptake (after 16 hrs at 37°C with FIP added following infection) were determined by surface staining for F protein (to detect surface bound virus; left panels) or GFP detection (which is only expressed on MV replication; right panels) by flow cytometry. The arrow marks the histogram for amitriptilyne treated cells. F protein expression or GFP fluorescence in uninfected cells are shown as filled histogram. C. Molt-4 or A549 cells were left untreated (black bars) or pre-exposed to amitriptyline prior to infection with Ed-eGFP (m.o.i. 2,5)(white bars). The percentage of GFP-expressing cells (left panel) and their mean fluorescent intensities (right panel) are indicated. One out of three representative experiments is shown.

To assess the overall impact of SMase activation on MV uptake into DCs, these were exposed to amitriptyline prior to infection. Thereby, intracellular accumulation levels of MV N protein 12 hrs following infection were reduced by about 50% (Fig. 4A, left panel) indicating that SMase activation is beneficial for viral DC infection. Consistent with this hypothesis, GFP levels produced from a tagged MV wild-type recombinant virus only on replication (IC323-eGFP) were reduced by about 50% upon siRNA mediated ablation of ASM expression (Fig. 4A, right panel). Pre-exposure to amitriptyline did not affect MV binding to DCs as determined by detection of MV F protein positive cells after 2 hrs at 4°C (Fig. 4B, left panel and graph), yet efficiently reduced intracellular GFP-accumulation after 24 hrs (with FIP added following infection to prevent MV spread) (Fig. 4B, right panel and graph). Amitriptyline did, however, not affect uptake or replication of a recombinant attenuated MV strain into T or epithelial cells (Fig. 4C) (which cannot be infected with IC323-eGFP due to the absence of CD150) indicating that SMase activation or ceramide elevation alone do not necessarily enhance MV infection as occurring in DCs.

### DC-SIGN signaling promotes CD150 surface recruitment

**Figure 5:**
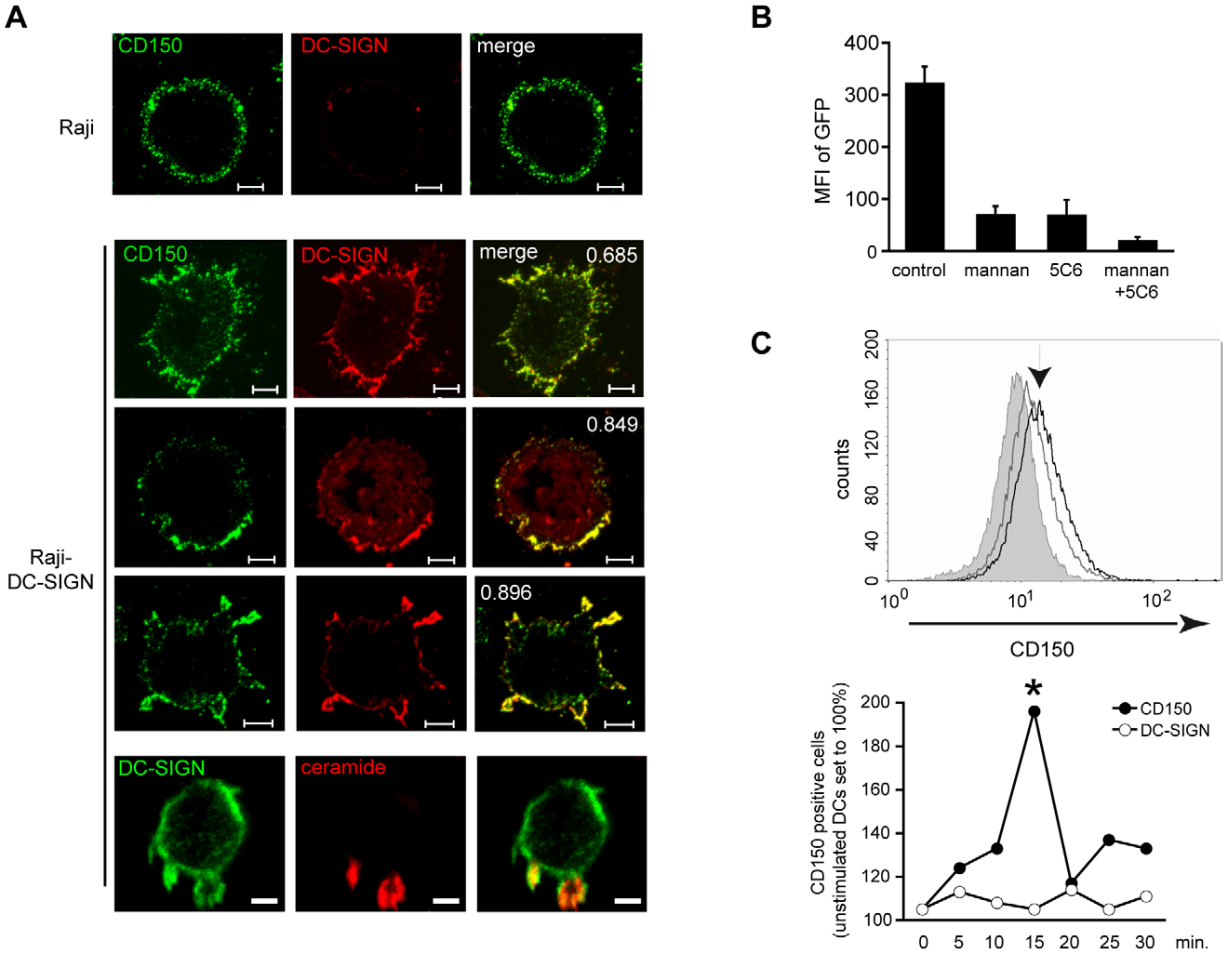
DC-SIGN ligation promotes surface redistribution of endogenous CD150. A. Raji (first row) or Raji-DC-SIGN cells (rows 2–5) were left untreated (rows 1 and 2) or exposed to AZ-D1 (followed by crosslink) for 5 (row 3) or 10 mins (rows 4 and 5), fixed and stained for CD150 (rows 1–4: green), DC-SIGN (rows 1–4: red, row 5: green) or ceramide (row 5: red). For this and the following experiments, colocalization coefficients were determined using the Pearson's algorithm (which ranges from −1 to +1, with values below 0,5 defined as no, between 0,5 and 0,75 as partial and above as high level of co-localization) and are indicated within the panels. Size bars represent 5 µm. B. DCs were left untreated or pre-exposed to mannan or a CD150-specific antibody (5C6) alone or in combination prior to infection with IC323-eGFP and subsequently cultured in the presence of FIP. The mfi of GFP-expressing cells was determined after 24 hrs. C. Surface CD150 was detected on DCs by flow cytometry. DCs were left untreated or exposed to mannan for 15 mins (upper panel, grey and black lines). The arrow marks mannan stimulated cells, the filled histogram shows the isotype control. Bottom graph: CD150 surface expression fluctuation in response to mannan stimulation withinn 30 mins. as compared to constant DC-SIGN levels. P<0,01.

**Figure 6:**
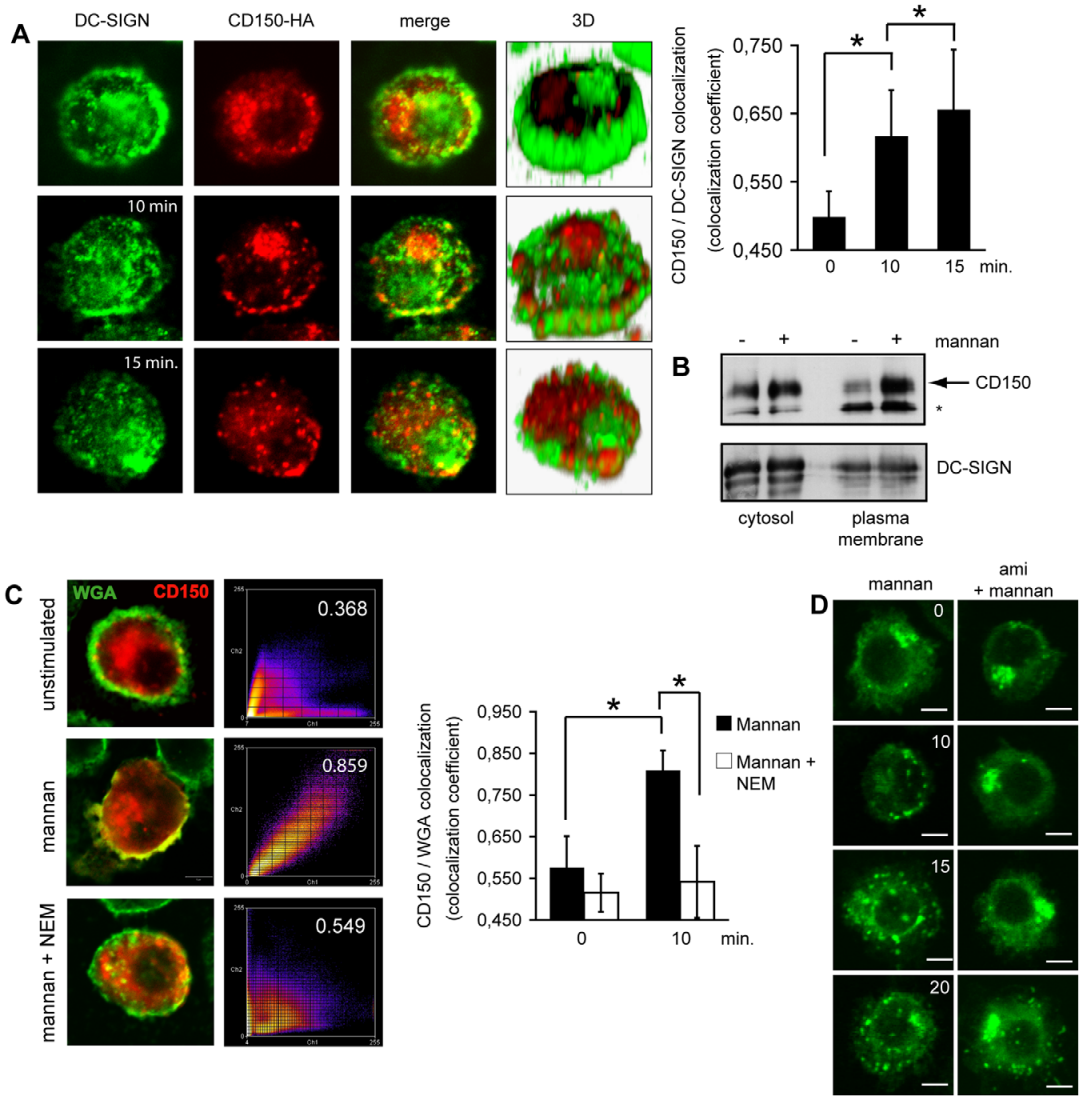
DC-SIGN ligation causes SMase dependent membrane redistribution of CD150 from intracellular compartments in DCs. A. DC-SIGN and CD150-HA were co-detected in DCs left untreated (upper panels) or exposed to mannan for 10 or 15 mins (middle and bottom panels). 3D reconstructions of z-stacks are shown for each condition. Colocalization coefficients for DC-SIGN and CD150 were determined and are indicared in the right graph (each 30 cells were recruited into the analysis). P = 0,01. B. CD150-HA transfected DCs were exposed to mannan or not, surface biotinylated and analysed for plasma membrane accumulation of CD150 (upper panel) or DC-SIGN (bottom panel) after cell lysis and precipitation using streptavidin beads (right lanes). Cytosolic detection of both proteins in lysates served as loading and transfection efficiency controls (left lanes). * represents a non-specific signal. C. CD150-HA and wheat germ agglutinin (WGA) were co-detected in DCs left untreated (upper panels) or pre-exposed for 15 mins to N-ethylmaleimide (NEM; bottom panels) or not prior to a 10 min. mannan treatment (middle panels). Colocalization coefficients were determined and are indicated within the right panels (which shown representative pseudocoloured scatter plots) or, for each 30 cells analyzed, in the right bar graph with standard deviations indicated. P>0,01. D. DCs transfected to express CD150-HA for 5 hrs were exposed to amitriptyline (right panels) or not (left panels) prior to mannan stimulation, fixed after the time intervals indicated (in mins) and stained for HA. Scale bars represent 5 mm.

**Figure 7:**
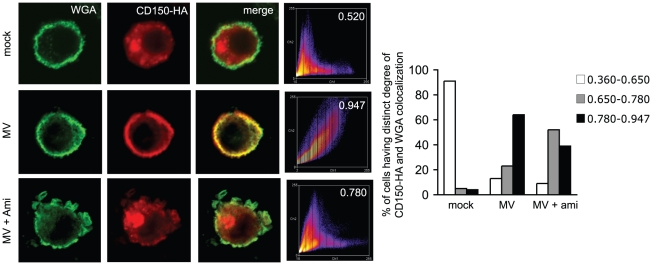
MV causes CD150 surface display in an amitriptyline sensitive manner. WGA and CD150 were codetected on CD150-DCs left untreated (upper row) or exposed to MV with (bottom row) or without a 2 hrs amitriptyline pre-treatment (middle row). Colocalization coefficients were determined (right intensity profiles) and are indicated for the examples shown within the panels, and for each 30 cells analyzed in the right graph where they were scored into no (white bars), intermediate (grey bars) or high (black bars) degree of WGA/CD150 co-segregation.

MV binding to DC-SIGN causes SMase activation on DCs which, in turn, promotes MV infection. We thus analysed whether this might relate to DC-SIGN-dependent alterations of membrane distribution of CD150. Because expression levels of this protein are low on DCs ([9], [10] and see below), we initially analysed the impact of DC-SIGN ligation on CD150 expression in Raji cells expressing high levels of endogenous CD150, and on stable transfection, DC-SIGN (Raji-DC-SIGN) (Fig. 5A, upper and second row). In untreated Raji-DC-SIGN cells, both molecules revealed a punctate expression pattern overall covering the cell surface (Fig. 5). DC-SIGN-ligation caused enhanced co-clustering of DC-SIGN and CD150 in large platforms (after 5 mins, Fig. 5A, third row), which subsequently protruded from the cell surface (after 10 mins, Fig. 5A, fourth row), revealing that DC-SIGN signaling indeed promotes redistribution of CD150. Suggesting a role of SMase activation in this process, DC-SIGN enriched protrusions emanating from the cell surface (prominent formation of which may relate to very low phospholipid scramblase levels of Raji cells [42]) were locally also enriched for ceramides (Fig. 5A, bottom row). As reported erlier, efficient MV uptake into DCs relies on both DC-SIGN (for trapping) and CD150 (particularly for fusion) [9], [10]. In line with earlier findings, interference of DC-SIGN interaction by mannan, an antibody or EGTA reduced MV binding to DCs by approximately 50% ([9] and Fig. S1), and blocking of either DC-SIGN and CD150 alone or in combination strongly interferes with MV uptake and replication (Fig. 5B). On immature DCs, expression of endogenous CD150 was generally low as reported [9], [10], yet increased surface display ligation within 15 mins after DC-SIGN was detectable by flow cytometry (Fig. 5C). To follow CD150 redistribution in response to DC-SIGN ligation in DCs in detail, we generated C-terminally HA-tagged CD150 which, when overexpressed in HeLa cells, did not differ with regard to subcellular distribution, surface expression level, glycosylation and DRM association from the unmodified protein (not shown). When transfected into DCs (CD150-HA-DCs), transgenic CD150, similar as the endogenous in DCs, mainly localized to intracellular compartments, while DC-SIGN, expectedly appeared in clusters at the cell surface [17], [19], [43] with little detectable overlap of both molecules (Fig. 6A, upper panels and right graph). Mirroring our findings in Raji-DC-SIGN cells, DC-SIGN ligation by mannan promoted both surface translocation, clustering of CD150-HA and co-clustering with DC-SIGN in DCs peaking between 10 and 15 mins after exposure (Fig. 6A, middle and bottom panels, and right graph) indicating that DC-SIGN-signaling indeed causes clustering and surface recruitment of this molecule. The latter was further confirmed by using a surface biotinylation/streptavidin precipitation approach with CD150-HA-DCs where exposure to mannan substantially increased the amounts of CD150 pulled down by streptavidin-beads (Fig. 6B, right lanes). The amounts of cytosolic CD150 were also slightly elevated on mannan exposure indicating that the CD150 storage compartment might reveal differential sensitivity to detergent lysis on DC-SIGN signaling (Fig. 6B, left lanes). DC-SIGN-dependent CD150-HA surface recruitment involved transport and membrane fusion of exocytic vesicles in a SNARE-dependent manner as indicated by its sensitivity to N-ethylmaleimide (Fig. 6C), and, also ASM activation as it was essentially abolished on pre-exposure to amitriptyline (Fig. 6D). Importantly, ASM inhibition also interfered with MV-induced CD150 surface recruitment as determined by WGA/CD150 co-segregation levels (Fig. 7).

### CD150 is co-transported with ASM to the cell surface

**Figure 8:**
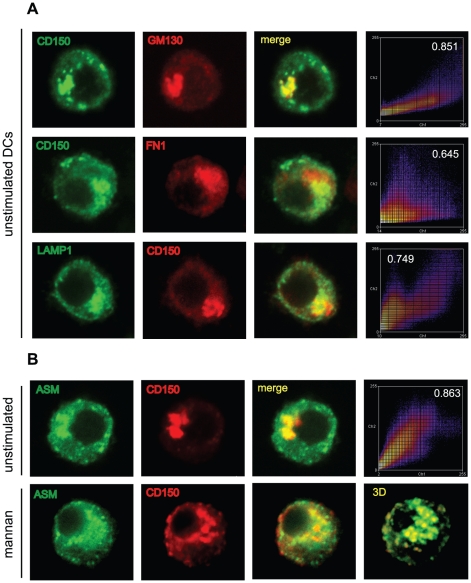
ASM is co-transported with CD150 from lysosomal compartments upon DC-SIGN ligation. A. The HA-tag was co-stained in CD150-HA transfected DCs with the cis-Golgi marker GM130 (upper panels), oligomerized MHC II class (FN1, second row), Lamp-1 (third row), and ASM (bottom row) and analyzed by laser confocal microscopy. Intensity profiles and colocalization coefficients are shown for the examples selected from a total of 30 cells analyzed in the right panels. B. CD150-HA expressing DCs were left untreated (upper panels 1–3) or stimulated with mannan for 10 mins (bottom panels) and analyssed for HA-tag and ASM expression by confocal microscopy. The bottom right panel shows 3D reconstruction of 20 z-stacks of mannan stimulated cell in the left panels, the upper right panel shows a representative profile example of CD150-HA/ASM colocalization with the coefficient indicated. A unstimulated cell has been chosen, each 30 unstimulated and mannan exposed CD150-DCs were recruited into the analysis.

To gain insight into the nature of the translocating CD150 compartment, we performed marker analyses in DCs transfected to overexpress CD150-HA. Expectedly, CD150-HA was co-detected with the trans-Golgi marker GM130 (Fig. 8A, upper row). CD150-HA does not accumulate in the MIIC loading compartment, as there is little co-segregation with oligomerized MHCII (detected by the FN1 antibody) (Fig. 8A, second row), yet rather in a Lamp-1 positive compartment that also contained ASM (Fig. 8A, third row). CD150 substantially colocalized with ASM in intracellular compartments in unstimulated DCs (Fig. 8B, upper row, first three panels), and both were redistributed to the cell surface on DC-SIGN ligation (Fig. 8B bottom row). Confirming co-segration of both molecules, the degree of colocalization remained identical prior to and after surface recruitment (an example for unstimulated DCs is shown in the pseudo-coloured scatter plot in Fig. 8B, upper row, right panel). These data indicate that CD150 shares an intracytoplasmic lysosomal compartment with ASM from which it is recruited to and displayed at the cell surface on DC-SIGN-mediated ASM activation to enhance MV entry into DCs.

## Discussion

As professional antigen-presenting cells, DCs operate at the interface of innate and adaptive immunity. Their location in the mucosa coins them first cells encountering invading pathogens also including viruses which, occasionally, exploit these cells as Trojan horses for transfer to secondary lymphoid tissues. They display a plethora of pattern recognition receptors (PRRs), and amongst those, the C-type lectin receptor DC-SIGN containing a mannose-binding domain has received particular attention with regard to its extraordinary pathogen–recognition capability which involves a broad panel of microorganisms and viruses also including HIV and MV. This interaction, especially for viruses, does not promote sorting into degradative compartments, but rather, DC-SIGN mediated enhancement of DC cis-infection or trans-infection of T cells have been described, and these involve enhanced access to the DC cytoplasm (as for MV [9], [10], or surface trapping of virions (as for HIV and CMV [44], [45], [46]) whereby they are concentrated and stored in invaginated compartments with plasma membrane continuity for subsequent transfer to conjugating target cells. In addition to these, signaling properties have been ascribed to DC-SIGN, which, though not able to initiate signaling pathways leading to regulated gene expression, modulates signals evoked after TLR ligation to stimulate NF-kB activation, and Rho-GTPase dependent activation of Raf-1 was found to be central [11], [21], [22].

SMase activation and subsequent ceramide accumulation were directly linked to c-Raf-1 and ERK activation in response to DC-SIGN ligation (Fig. 2B) and this in line with previous observations made in other cell types. Thus, the ability of KSR1, identified as essential component of the DC-SIGN signalosome [21] to activate Raf-1 and to enhance its activity towards ERK1/2 requires recruitment and specific binding to ceramide-enriched platforms in Cos-7 and intestinal epithelial cells [33], [34], [35], [36], [37]. In addition, Raf-1 activation in response to ceramide activation and physical interaction of c-Raf-1 with ceramides in response to IL-1β in mesangial cells were described [47], [48]. Though our findings with regard to the importance of DC-SIGN signal initiation are in agreement with these observations, those elaborating on the role of DC-SIGN modulation of TLR signaling are obviously not. As shown by two independent methods, we were unable to confirm an enhancing effect of DC-SIGN signaling on TLR-induced NF-κB activation [11] using either antibodies (the ability of which to promote c-Raf or ERK activation has been documented by us (Fig. 2B) and others [11], [22], [41] or mannan at any time point analysed (Fig. 3). The reasons for this discrepancy remain unknown, yet are not likely to include donor dependent variations, or any obvious technical problems since the reagents used gave reliably the expected positive results in control experiments (e. g. N
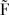
κB DNA binding and nuclear translocation in response to TLR signaling alone (Fig. 3) or the ability of antibodies or mannan to promote DC-SIGN activation (Figs. 2B)). Because abrogation of SMase activation rather promoted LPS-induced N
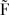
κB activation, our data suggest that DC-SIGN signaling may weaken rather than enhance TLR4 signaling (Fig. 3 A,B), thereby downregulating inflammatory responses. This hypothesis is in line with ASM dependent downregulation of LPS-induced TNF-α production in macrophages [49], and suggest that SMase activation in response to a PRR such as DC-SIGN might be an efficient regulator of systemic inflammatory responses.

SMase induction and subsequent membrane ceramide accumulation on DC-SIGN ligation reveals a kinetics typically observed for other receptors activating this pathway as well [27]. Both NSM and ASM are activated by DC-SIGN (Fig. 1C and D), and interestingly, this, though DC-SIGN-independently, also occurred in T cells exposed to MV [50] or on ligation of TNF-R or IL-1β-R [51], [52]. As evidenced from their kinetics of induction and the ability of both amitriptyline and GW4896 to interfere with DC-SIGN-dependent NF-κB modulation (Fig. 1 and 3B), NSM and ASM might be induced sequentially as reported by us in T cells previously [50] which, was, however, not further addressed in the present study. It is, however, tempting to speculate that NSM activation might promote redistribution and plasma membrane fusion of a lysosomal Lamp1+ compartment containing both ASM and CD150 (Fig. 8) in an exocytic process which, due to its NEM sensitivity, involves as yet unidentified SNAREs (Fig. 6C).

Their activation by an established DC-SIGN agonist (and sensitivity to EGTA inhibition) and cross-linked specific antibodies clearly links SMases and ceramides to DC-SIGN ligation (Fig. 1). MV is known to interact with other signaling surface receptors on DCs which, theoretically, could also elicit these responses. CD46 is unlikely to be involved, since wild-type MVs as that used in this study do not interact with this receptor [53]. MV binding to CD150, which is expressed to low levels on DCs (Fig. 5B and [9], [10]), is not known to depend on divalent cations (neutralisation of which abrogate ceramide induction by MV (Fig 1A)) nor do antibodies directed against this molecule prevent their MV-induced activation (Fig. 1A) indicating that CD150 also does not contribute. MV is known to act as TLR2, but not TLR4 agonist on monocytes, and LPS or PamCSK dependent upregulation of CD150 has been proposed to support MV infection of these cells [54], [55]. Though LPS signaling in various cell types also including DCs can involve ASM, this needs, however, tight regulation as it results in massive DC apoptosis if high doses of LPS are applied [56] or, on standard dose LPS application (100 ng/ml), if ceramide turnover is prevented [57], [58]. Ceramide generation, however, is insufficient to promote most responses to LPS including NF-κB activation [59], and in line with these observations, DC-SIGN ligation alone does not activate NF-κB [11] and SMase inhibition by amitriptyline did not interfere with LPS-driven upregulation of CD83 and CD86 in our system as well (Fig. 2A). Ligation of TLR2 by MV, not yet directly shown to occur on DCs so far, is unlikely to contribute to early ASM induction which is EGTA sensitive (Fig. 1), may, however, contribute to late upregulation of CD150 (as measured in monocytes late (12 or 24 hrs) after TLR2 stimulation [54], [55]) coincided with that of proinflammatory cytokines and may thus occur secondary to IL-1R ligation by IL-1β [7], a well established SMase activator [51], [60].

SMase activation as induced upon DC-SIGN ligation is beneficial for MV uptake into DCs (Fig. 4), and this may also be supported by biophysical properties of ceramide-enriched domains such as promotion of negative membrane curvature which can favor receptor mediated endocytosis [28], [39], or their gel-like phase supporting membrane fusion in general [27], [40]. As these also apply to SMase activators other than DC-SIGN, recruitment and segregation of specific receptors into these platforms may be decisive for their respective role in pathogen uptake. For MV, DC-SIGN clearly enhances DC *cis*-infection [9], [10], and this is greatly aided by rapid surface recruitment of CD150 which co-clusters with DC-SIGN. Thereby, SMase activation promotes surface translocation and compartimentalization of a receptor promoting MV fusion (Fig. 6 and 7). Interestingly, MV binding to DCs is not strengthened on SMase activation (Fig. 4B) indicating that major trapping of MV relies on DC-SIGN. This corroborates our earlier observations that DC maturation (known to upregulate CD150 [9], [10]) does not increase MV binding to these cells [61]. In line with DC-SIGN acting as a major trapping factor for MV it is not surprising that pre-ligation of this molecule by crosslinked DC-SIGN antibodies or mannan blocks rather enhances MV uptake into these cells because they render DC-SIGN inaccessible to MV binding (Fig. 5B). Surface recruitment and compartimentalization of CD150 may act in concert with the fusion promoting membrane environment provided by SMase activation [40] to support viral entry. If entry receptors are, however, highly abundant (such as CD46 on Molt-4 or A549 cells), SMase activation may not have substantial effects on viral uptake (Fig. 4C). For HIV, however, ceramide induction elevation even acts antivirally, since it shifted the virus into endocytic, degradative uptake route in phagocytic cells, and prevented lateral co-segregation of CD4 and CXCR4 and thereby membrane fusion [62], [63], [64]. In DCs, where HIV is not routed into a degradative compartment on DC-SIGN interaction, ceramide interference with receptor co-segregation would be consistent with compartimentalization and storage of virus for *trans*-infection of T cells which is efficiently prevented on ablation of DC-SIGN interaction. It will thus be interesting to determine if segregation of receptors promoting uptake and routing of pathogens in response to SMase activation in DCs by DC-SIGN and other stimuli follows a common or counter-regulatory role which may essentially decide the outcome of the interaction of a given pathogen with these cells.

Most interestingly, CD150 has recently been identified as microbial sensor on macrophages essentially promoting phagosome routing and recruitment of the cellular machinery for bacterial killing [65]. If this would apply to DCs, upregulation of CD150 in an SMase dependent manner might directly impact on routing of pathogens other than MV in DCs.

## Materials and Methods

### Ethics statement

Primary human cells were obtained from the Department of Transfusion Medicine, University of Würzburg, and analysed anonymously. All experiments involving human material were conducted according to the principles expressed in the Declaration of Helsinki and ethically approved by the Ethical Committee of the Medical Faculty of the University of Würzburg.

#### Cells and virus infection

Immature DCs were generated from monocytes enriched from peripheral blood obtained from healthy donors by Ficoll gradient centrifugation followed by plastic adherence and subsequent culture in RPMI1640 containing 10% FCS and human GM-CSF (500U/ml, Berlex, Germany)/IL-4 (250U/ml, Miltenyl Biotec, Germany) for 3 to 6 days. If not stated otherwise, DCs were used immature for all experiments. B lymphoblastoid Raji, Raji-DC-SIGN cells (kindly provided by T. Geijtenbeek, Amsterdam) and Molt-4 cells were maintained in RPMI1640/10% FCS, A549 cells in MEM 5% FCS. MV wild-type strain WTF, the MV recombinants IC-323-eGFP (wild-type) (based on the wild-type strain IC-323, kindly provided by Y. Yanagi, Fukuoka), ED-eGFP (attenuated) (kindly provided by P. Duprex, Belfast) and MGV (expressing VSV G protein instead of the MV glycoproteins [66] (all grown on human lymphoblastoid BJAB cells in RPMI1640/10% FCS) were titrated on marmoset lymphoblastoid B95a cells (kept in RPMI1640/5%FCS). For exposure experiments, MVs grown on BJAB cells were purified by sucrose gradient ultracentrifugation as was the MOCK control from uninfected BJAB cells. For binding experiments, DCs were exposed to MVs (if not indicated otherwise, multiplicity of infection 2,5) for 2 hrs at 4°C. A fusion inhibitory peptide (FIP) Z-D-Phe-L-Phe-Gly-OH (Bachem, Heidelberg, Germany; 200 mM in DMSO) was included in these and infection experiments a 2 hrs after exposure.

#### Ceramide/SMase detection

For surface detection of ceramides and ASM we adopted an assay previously described [67]. Briefly, cells (each 3×10^5^) were stimulated for the time intervals indicated. α-DC-SIGN monoclonal antibody AZ-D1 (kindly provided by T. Geijtenbeek, Amsterdam) (1 mg/ml; if not stated otherwise, pre-ligated for 15 mins on ice by 10 mg/ml gout-a-mouse antibody), MOCK, MV or mannan (1mg/ml, Sigma Aldrich) were used for DC stimulation. Cells were fixed in 1% formaldehyde and incubated with α-ceramide IgM (clone MID 15B4, Alexis), a polyclonal rabbit α-ASM IgG (H-181; Santa Cruz) over night at 4°C and washed extensively in PBS. Cell bound antibody was desorbed for 30 sec with ice cold 100 mM glycine-HCl, pH 2.5, and spotted onto nitrocellulose following neutralisation with 100 mM Tris-HCl pH 8.0. Antibodies were detected by goat-α-mouse IgM conjugated to peroxidase (Dianova, Hamburg, Germany) and chemiluminescence was quantified using AIDA software (Raytest, Straubenhardt, Germany). Alternatively, cell surface ceramides were detected on fixed DCs using flow cytometry analysis after incubation with α-ceramide antibodies (1∶30 dilution) at 37°C for 1h and Alexa-488-conjugated secondary α-mouse IgM (1∶200 dilution, MolecularProbes) at 4°C for 30 mins. Alternatively, ASM or NSM enzymatic activities were determined using a commercial assay kit according to the manufacturers instructions (Amplex Red Sphingomyelinase assay kit, Invitrogen). Membrane enriched fractions were used for NSM activity detection [27], [40]. When indicated, the paired student's t-test was used for statistical analysis.

### CD150 expression vector, siRNA tranfection and RT-PCR analysis

CD150-HA was generated by PCR mediated HA-tag insertion at the C-terminus of CD150 full-length cDNA and cloning under CMV promoter in pCG vector. 15 µg of plasmid were nucleofected into 2×10^6^ DCs following the manufacturer's protocol (Amaxa). For silencing of NSM2, DCs were transfected with 100 nM siRNA targeting human *SMPD3* (NSM2) specific [68] or, for control, a scrambled siRNA (Eurogentec, Belgium) using transfection reagent DF4 (Dharmacon, Lafayette, CO), according to the manufacturer's protocol. Before cells were recruited into the respective experiments (after 72 hrs), aliquots were harvested for RNA extraction (Qiagen, RNAeasy Kit) and subsequent RT-PCR analyses. Forward 5′-GCCCTTATCTTTCCATGCTACTG-3′ and reverse 5′-ACAGAGGCTGTCCTCTTAATGCT-3′ primers were used for specific *SMPD3* amplification.

#### Biotinylation, immunoprecipitation and Western blot analysis

3×10^6^ CD150-HA transfected DCs were stimulated for 15 mins with 1mg/ml mannan, washed twice with ice cold PBS, pH 8.0 and rotated with 0,5 mg/ml Sulfo-NHS-LC-Biotin (Pierce) for 30 mins at room temperature, washed with PBS, 100 mM Glycin, pH 7,0 and lysed in buffer containg 1% Triton-X 100. Biotinylated surface proteins were precipitated using streptavidin beads (Pierce) overnight at 4°C and subjected to Western blot analysis for CD150 and DC-SIGN expression using rabbit polyclonal antibodies directed against the HA-tag (Y-11) or DC-SIGN (H-200, both Santa Cruz).

Extracts isolated from phorbolester/ionomycin (PMA 40ng/ml, ionomycin 2,5 mM, Sigma Aldrich) or α-DC-SIGN activated DC cultures were harvested and analysed using antibodies directed against p-c-Raf-1 (Ser338), ERK, and pERK (Thr202/Tyr204) (Cell Signalling, Frankfurt, Germany) in Western blot. When indicated, chemiluminescence was quantified using the AIDA software program (Raytest, Straubenhardt, Germany).

#### N
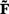
kB activation assays

DNA binding activity of NF-κB p65 was determined with ELISA based TransAM NF-κB family kit (Active Motif, Carlsbad, CA), according to the manufacturer's protocol. Experiments were repeated three times using cells of different donors.

#### Flow cytometry and immunostaining

Cell surface levels of ceramides, CD83, CD86 and CD150 were detected by flow cytometry by staining with antibodies specific for ceramides (clone MID 15B4, Alexis), CD83 and CD86 (BD Biosciences Pharmingen), CD150 (5C6). Intracellular levels of MV N protein were determined using a specific antibody (F227, generated in our laboratory). Alternatively, GFP was detected in cells infected with IC-323-eGFP or ED-eGFP. For immunostaining, DCs (when indicated pre-exposed for 2 hrs to amitriptyline (10 mM), GW4869 (1,3 µM), or 15 mins to N-ethylmaleimide (NEM, 1mM) (all Sigma-Aldrich, Taufkirchen, Germany) were transferred onto 8-chamber slides (LabTekII, Nunc, Wiesbaden, Germany) pre-coated with poly-L-lysine and subsequently activated by LPS (100 ng/ml), mannan (1mg/ml), DC-SIGN-specific polyclonal antibody H200 (Santa Cruz) or mannan for the time intervals indicated at 37°C. For immunostaining, cells were fixed in paraformaldehyde (4% in PBS) and stained for membrane ceramide (clone MID 15B4, Alexis) or, after permeabilisation (0.1% Triton X-100) for CD150 (clone IPO-3, Abcam), oligomerised MHC class II (FN-1; kindly provided by Steinar Funderud), Lamp-1 (rabbit polyclonal serum; kindly provided by Soren Carlsson, Umea, Sweden), p65 (C22B4, Cell Signalling, Frankfurt, Germany), wheat germ agglutinin or ASM (H181, Santa Cruz). Actin was detected using Alexa 594 conjugated phalloidin (Molecular Probes, Karlsruhe, Germany), DAPI was used to stain nuclei. Fluorochrome G (Southern Biotech, Eching, Germany) mounted samples were analysed by confocal laser scanning microscopy (Laser Scan Microscope, LSM510 Meta, Software version 3.2, SP2; Axiovert 200M microscope, Objective: 63×; aperture 1.4 plan apochromat; when indicated, vertical z-stacks were acquired (20 optical planes) and 3D deconvolutions were performed (by using Zeiss software). When indicated, colocalization coefficients were determined using the Pearson's algorithm (which ranges from −1 to +1, with values below 0,5 defined as no, between 0,5 and 0,75 as partial and above as high level of co-localization). The pseudo-coloured scatter plots shown display frequencies of the red-green pixels in the original images. Hot colours resresent high values of colocalization.

## Supporting Information

Figure S1 DC-SIGN blocking interferes with MV binding to DCs. DCs were pretreated with mannan at the concentrations indicated, a DC-SIGN-specific antibody (H200) (10 µg/ml) or 10 mM EGTA prior to MV exposure (m.o.i. 2). Percentages of cells staining for MV F protein and mfis were determined by flow cytometry following a 1hrs incubation period on ice.
(0.45 MB EPS)
